# Electric Dipole Coupling of a Bilayer Graphene Quantum Dot to a High-Impedance Microwave Resonator

**DOI:** 10.1021/acs.nanolett.4c01791

**Published:** 2024-06-04

**Authors:** Max J. Ruckriegel, Lisa M. Gächter, David Kealhofer, Mohsen Bahrami Panah, Chuyao Tong, Christoph Adam, Michele Masseroni, Hadrien Duprez, Rebekka Garreis, Kenji Watanabe, Takashi Taniguchi, Andreas Wallraff, Thomas Ihn, Klaus Ensslin, Wei Wister Huang

**Affiliations:** † Laboratory for Solid State Physics, ETH Zürich, CH-8093 Zürich, Switzerland; ‡ Quantum Center, ETH Zürich, CH-8093 Zürich, Switzerland; § Research Center for Electronic and Optical Materials, National Institute for Materials Science, 1-1 Namiki, Tsukuba 305-0044, Japan; ∥ Research Center for Materials Nanoarchitectonics, National Institute for Materials Science, 1-1 Namiki, Tsukuba 305-0044, Japan

**Keywords:** Quantum dots, bilayer graphene, van der Waals materials, dispersive coupling, microwave resonator

## Abstract

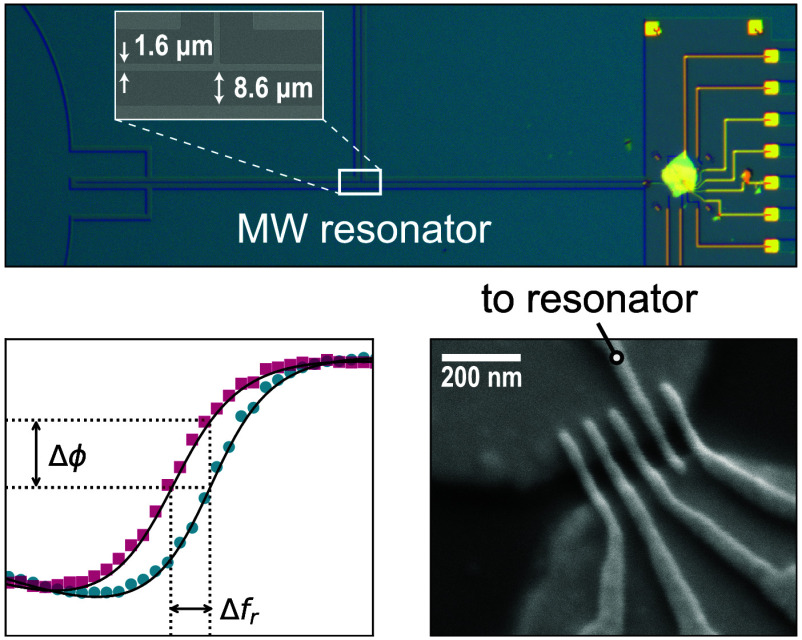

We implement circuit quantum electrodynamics (cQED) with quantum dots in bilayer graphene, a maturing material platform that can host long-lived spin and valley states. Our device combines a high-impedance (*Z*_r_ ≈ 1 kΩ) superconducting microwave resonator with a double quantum dot electrostatically defined in a graphene-based van der Waals heterostructure. Electric dipole coupling between the subsystems allows the resonator to sense the electric susceptibility of the double quantum dot from which we reconstruct its charge stability diagram. We achieve sensitive and fast detection of the interdot transition with a signal-to-noise ratio of 3.5 within 1 μs integration time. The charge–photon interaction is quantified in the dispersive and resonant regimes by comparing the resonator response to input–output theory, yielding a coupling strength of *g*/2π = 49.7 MHz. Our results introduce cQED as a probe for quantum dots in van der Waals materials and indicate a path toward coherent charge–photon coupling with bilayer graphene quantum dots.

The canonical circuit quantum electrodynamics (cQED) system is a solid-state qubit coupled to photons in a superconducting microwave resonator.^1^ While the large dipole moments common to many superconducting qubits enable strong coupling,^2^ even the minuscule dipole of an individual electron isolated in a semiconductor quantum dot (QD) can interact coherently with microwave photons.^3,4^ Strong coupling enables applications such as fast charge and spin readout,^5^ high-resolution state spectroscopy,^6^ and photon-mediated long-range interactions.^7−9^ Combining cQED with semiconductor QDs is therefore of practical relevance to a possible spin qubit device architecture.^10^ Hybrid cQED has been realized in semiconductor materials like GaAs,^3,11^ silicon,^12−14^ germanium,^15,16^ and InAs.^17,18^ In this work, we extend this list to van der Waals (vdW) heterostructures by demonstrating hybrid cQED experiments with QDs in bilayer graphene.

Making use of the variety of vdW materials in cQED is a promising route for quantum technologies. For example, highly coherent quantum circuits built with hexagonal boron nitride (hBN) and NbSe_2_ have been reported.^19,20^ Also monolayer graphene has been integrated with microwave circuits in the form of etched nanostructures.^21,22^ Here, we choose as our material bilayer graphene encapsulated with hBN, whose voltage-induced bandgap and atomically clean interfaces make it an excellent 2D material for electrostatically defined QDs.^23^ Moreover, as a host for spin qubits, bilayer graphene also promises low spin decoherence rates due to its weak spin–orbit coupling^24,25^ and weak hyperfine interaction.^26^ Because of these properties, bilayer graphene has become a rapidly developing material platform for spin and valley qubits. Crucial ingredients for quantum computing applications, including time-resolved charge detection^27,28^ and switchable Pauli spin and valley blockade,^29−31^ have been demonstrated in electrostatically defined bilayer graphene QDs. Spin lifetimes of up to 400 ms in these systems^32,33^ are comparable to those of other semiconductor QD systems like III–V,^34−36^ silicon-^37−40^ and germanium-^41^ based heterostructures. Bilayer graphene also offers a controllable valley degree of freedom, which exhibits exceptionally long lifetimes approaching 1 s.^42^ The long relaxation times allow for high-fidelity spin and valley qubit readout.^32,42^ However, the detection bandwidth of the dc charge sensors has so far been limited to around 2 kHz.^43^ These characteristics make gate-based dispersive charge readout^44,45^ with microwave resonators attractive for bilayer graphene.

In this paper, we present a device that integrates an on-chip superconducting resonator with a bilayer graphene double QD (DQD). We use the kinetic inductance of NbTiN to realize a high-impedance resonator whose zero-point voltage fluctuations are more than four times larger than for a conventional 50 Ω resonator. The larger voltage fluctuations enhance the coupling strength *g* to the electric dipole moment of DQD state transitions. We demonstrate resonator-based sensing of the DQD electric susceptibility, complementing transport measurements. The high sensitivity of the measurement allows us to detect an interdot charge transition with a signal-to-noise ratio (SNR) of 3.5 within 1 μs integration time, a charge sensitivity comparable to state-of-the-art measurements of a double quantum dot in silicon with on-chip resonators.^5^ We estimate the minimum time *t*_min_ ≈ 1 × 10^–7^ s needed to achieve a SNR larger than one, 4 orders of magnitude faster than current dispersive gate-based sensing of QDs in bilayer graphene.^45^ Furthermore, we measure the resonator response to the DQD dipole moment in both the dispersive and resonant cases, and extract the relevant system parameters from a least-squares fit to input-output theory for quantum dots.^46^ We achieve a bare resonator coupling strength of up to *g*/2π = 49.7 MHz that we assume to be limited by the small lever arm difference β of the coupling gate. The charge decoherence rate γ = 643 MHz is likely caused by high tunnelling rates of electrons to the leads, effectively making this value an upper bound for γ.^6,47^ This work constitutes the next technological step for bilayer graphene as a spin or valley qubit platform and marks the necessary advances to achieve coherent charge-photon coupling in vdW materials.

Figure 1 shows the hybrid device. We sputter 15 nm of NbTiN on an intrinsic silicon wafer with 100 nm SiO_2_. A half-wavelength resonator is patterned into the superconducting film as a coplanar waveguide with a center conductor width of 1.6 μm and a length of 900 μm [panel (a)]. Based on the resonator geometry and its center frequency *f*_r_ = 6.033 GHz we estimate the resonator impedance  1 kΩ. One end of the resonator is capacitively coupled to a 50 Ω feedline. Close to the other end, we fabricate the bilayer graphene DQD device [see schematic in panel (b)]. The vdW material stack consists of Bernal-stacked bilayer graphene, encapsulated in hexagonal boron nitride (hBN) and back-gated with graphite. Two layers of gold gates are fabricated on top of the vdW material stack with ALD-deposited Al_2_O_3_ as intergate dielectric. One of the gates connects to the resonator center conductor. We apply a voltage to this gate through a quarter-wavelength bias tap at the midpoint of the resonator. Electron transport through the quantum dot is probed via ohmic contacts to the graphene, achieved by etching through the top layer of hBN before metal deposition. To prevent microwave leakage through parasitic gate capacitances,^48,49^ all gate lines are biased through on-chip low-pass filters (see Supporting Information for details).

**Figure 1:**
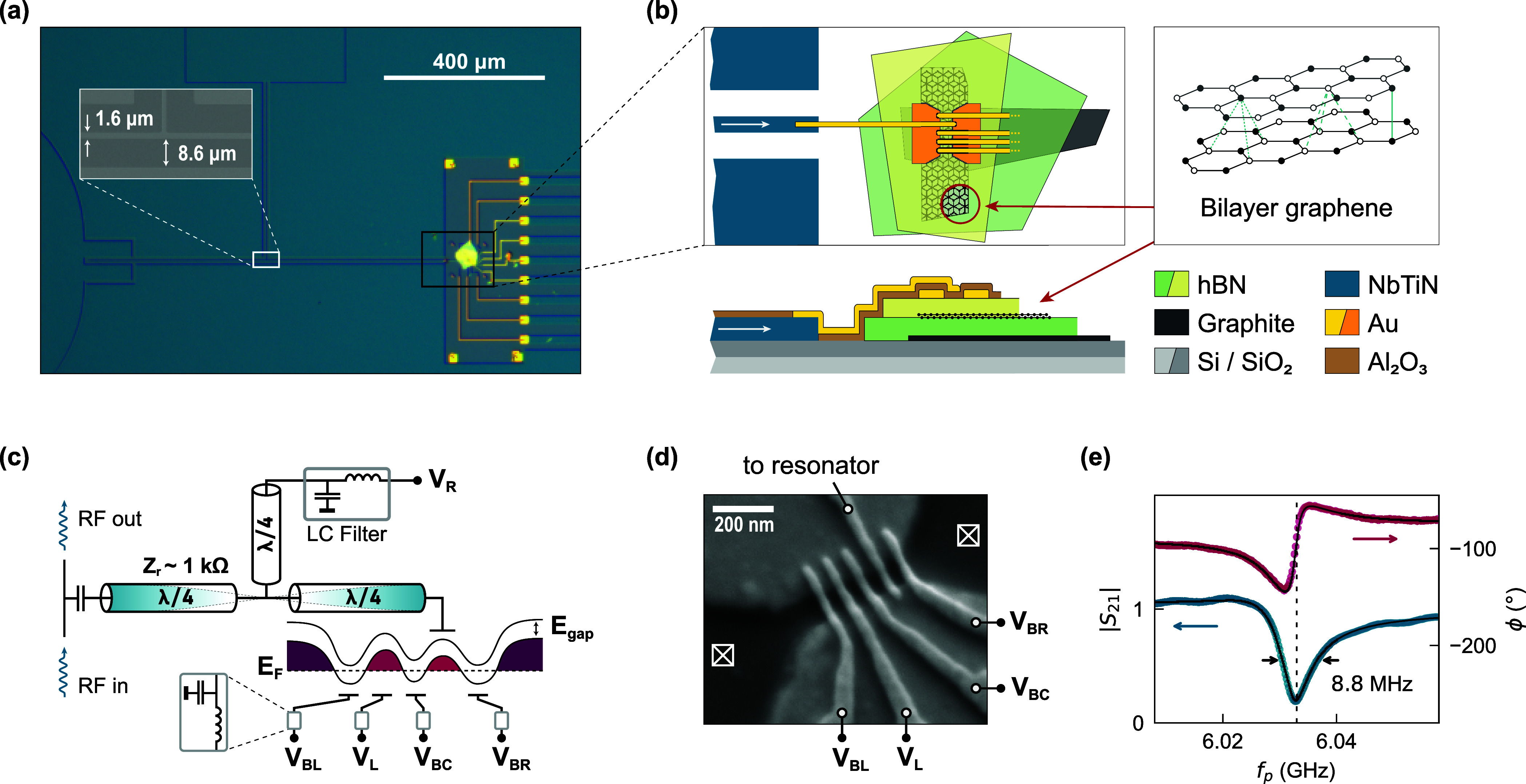
Bilayer graphene double quantum dot (DQD) integrated with a high-impedance microwave resonator. (a) Optical image of the device showing the microwave circuit fabricated from 15 nm thick NbTiN with the vdW material stack on the right (black rectangle). Inset: Scanning electron micrograph (SEM) of the coplanar waveguide (CPW) at the midpoint of the half-wavelength resonator. (b) Top view and cross-section schematic of the DQD device. The vdW material stack with bilayer graphene, encapsulated in hBN and back-gated with graphite, is deposited on a Si/SiO_2_ substrate. Two layers of metal top gates define the DQD device. (c) A simplified circuit schematic of the device in (a). The resonator is probed via a feedline (left). The voltage *V*_R_ is applied to the plunger gate through the center conductor of the CPW via a LC-filter and quarter-wavelength tap. The Fermi energy *E*_F_ of the bilayer graphene along the channel is tuned by plunger and barrier gates to form a double-well potential for holes. (d) SEM of the gates on top of the vdW material stack, with voltages applied to the barrier and plunger gates labeled. Crossed boxes indicate ohmic contacts to the bilayer graphene. (e) Microwave transmission spectrum through the feedline around the resonator frequency *f*_r_ (dashed line).

The sample is mounted in a dilution refrigerator that reaches the base temperature *T* = 10 mK. For resonator spectroscopy, we probe the microwave transmission through the feedline as a function of frequency *f*_p_. The signal undergoes a heterodyne detection scheme that allows us to measure the complex transmission amplitude *S*_21_(*f*_p_) = *Ae*^*iϕ*^ = *I* + *iQ*. From a fit^50^ to the spectrum around *f*_r_ [see Figure 1 (e)] we extract the internal resonator decay rate κ_int_/2π = 1.9 MHz and the total line width κ/2π = 8.8 MHz. The internal quality factor *Q*_int_ = 2*πf*_r_/κ_int_ = 3250 is limited by the dielectric environment of SiO_2_ and Al_2_O_3_,^51^ but is sufficiently high to not dominate the total quality factor.

A negative voltage applied to the back-gate opens a bandgap in bilayer graphene and together with a pair of split-gates confines valence band holes to a narrow channel. The second set of gates across the channel defines plunger and barrier gates [see Figure 1(c) and (d)]. The plunger gate voltages *V*_L_ and *V*_R_ change the electrochemical potentials of electrons in the left and right QD. The gate voltages *V*_BL_, *V*_BC_, and *V*_BR_ are set to place the Fermi energy in the bandgap such that these regions act as tunnel barriers. The gate connected to the resonator is the right QD plunger gate, biased through the resonator and low-pass filter with *V*_R_. Its capacitance to the right QD couples the DQD to the electric field fluctuations at the voltage antinode of the microwave resonator. This makes the resonator susceptible to the dipole moment of electrons in the QDs and thereby acts as a sensor.

Figure 2(a) and (b) depict the DQD charge stability diagram measured simultaneously in dc current and resonator response. The source-drain current *I*_SD_ through the device as a function of plunger gate voltages *V*_L_ and *V*_R_ shows the hexagonal pattern typical for transport through a DQD. Within one hexagon, the number of charges in each QD is constant, and the current is suppressed due to Coulomb blockade. At the four boundaries of a charge configuration, indicated by dashed lines in (a), the electrochemical potential of one QD is resonant with the Fermi energy of the respective lead while the other dot is Coulomb blockaded, and transport can occur via cotunnelling processes through the other dot. High current at the triple points, marked with circles, corresponds to the QD electrochemical potentials in both dots being aligned inside the bias window.

**Figure 2:**
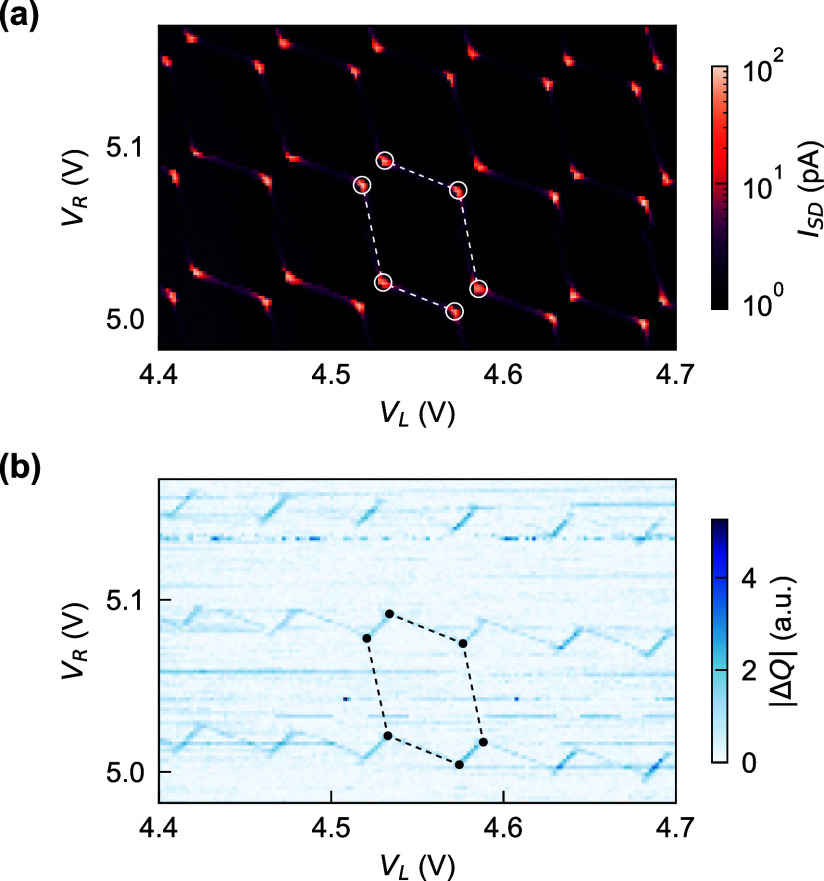
DQD charge stability diagram. (a) Current *I*_SD_ measured through the channel at a 200 μV bias voltage as a function of plunger gate voltages. White dashed lines mark the boundaries of one hexagonal region with stable charge configuration. Circles mark the triple points. (b) Change in the quadrature component *Q* of the microwave signal transmitted through the feedline at fixed probe frequency *f*_p_ = *f*_r_. The charge stability diagram is visible (marked in black) with interdot transitions connecting the triple points.

Simultaneous with the current, we monitor the complex microwave transmission through the feedline with the probe frequency *f*_p_ = *f*_r_. Figure 2(b) shows the change in the quadrature component *Q* of the complex signal as a function of *V*_L_ and *V*_R_. The observed pattern matches the current data with a change in *Q* along the lines of positive slope connecting adjacent triple points and lines of negative slope coinciding with the cotunnelling current visible in (a). Along these cotunnelling lines, the resonator is susceptible to tunnelling of electrons between the right QD and its lead. The signal between adjacent triple points corresponds to transitions between charge configurations with the same total number of electrons. The charge states hybridize along interdot transitions due to the tunnel coupling *t*_c_ between the QDs. These hybridized states form a two-level system whose electric susceptibility changes the resonator response and therefore the transmission through the feedline at *f*_p_.

We observe this sensing signal over a wide range of gate voltages and QD configurations. Figure 3(a) shows an interdot transition at higher gate voltages with a strong phase response. Later we will show that this sensing signal of the DQD electric susceptibility is in the dispersive regime. Sweeping *V*_L_ across the transition (black arrow) changes the energy detuning δ between the QD electrochemical potentials in the left and right dot. Microwave spectroscopy at two different QD detunings [Figure 3(b)] reveals a clear shift of the resonator to lower frequencies for δ = 0 (pink squares) compared to nonzero detuning (blue circles). Measured at a fixed-frequency probe tone *f*_p_, the shift of the resonator frequency results in a phase change Δϕ of more than 26°.

**Figure 3:**
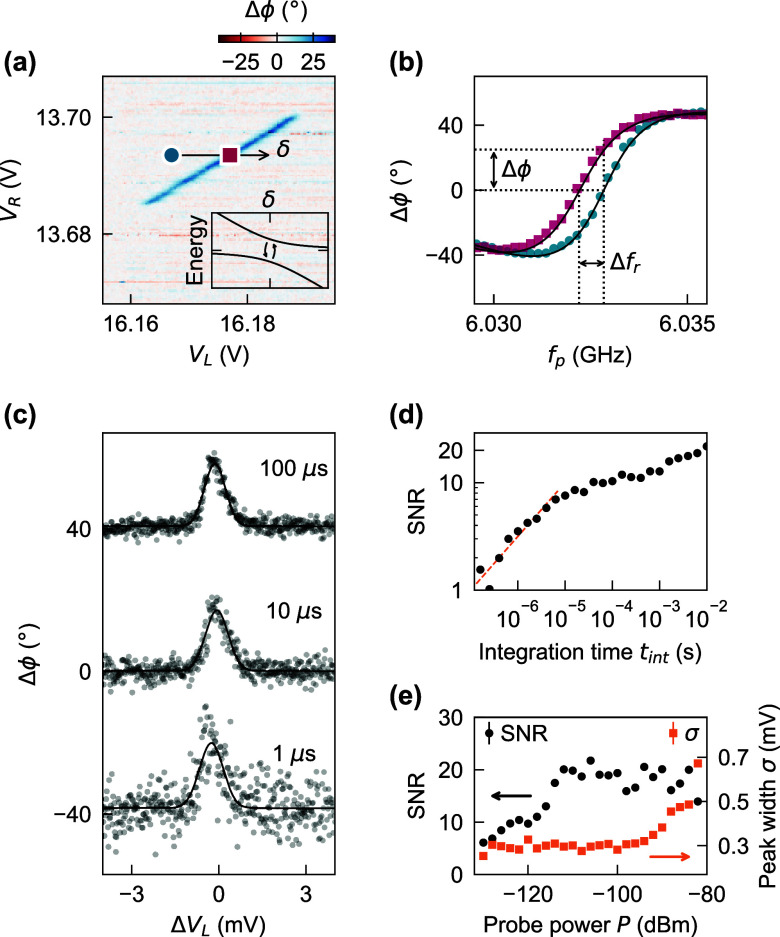
Dispersive resonator shift and SNR analysis. (a) Microwave response of one example interdot transition. The black arrow indicates the detuning axis. The inset is a schematic energy diagram of the charge qubit states as a function of QD energy detuning δ. Tunnel coupling leads to hybridization at δ = 0 with an energy splitting 2*t*_c_. (b) Phase response of the resonator as a function of probe tone *f*_p_ at the two detunings marked by symbols in (a). Dispersive interaction with the charge qubit shifts the resonator to lower frequency at δ = 0 (pink squares) compared to δ = 4 mV (blue circles). (c) Three example traces across the transition for different integration times *t*_int_. A fit of a Gaussian peak to the data (black solid lines) determines the SNR as a function of *t*_int_. For *t*_int_ = 1 μs the phase signal is still clearly discernible with a SNR of 3.5. (d) The SNR reduces with shorter *t*_int_ but stays well above unity even for integration times below 1 μs. A linear extrapolation to SNR = 1 (orange solid line) gives *t*_min_ ≈ 1 × 10^–7^ s. (e) SNR and width σ of the fitted Gaussian peak as a function of resonator probe power. Error bars for SNR in (d) and (e) are smaller than the marker size.

The detection signal is investigated in more detail by determining the signal-to-noise ratio (SNR) as a function of integration time and probe power. We sweep the gate voltage *V*_L_ across the interdot transition and reduce the measurement integration time *t*_int_ from 100 ms to 100 ns at a probe power of *P* = −90 dBm at the sample. Figure 3(c) shows three examples of measured traces for different integration times. A fit of a Gaussian function to the data (solid lines) determines the peak height *A* and the peak width σ. Subtracting the fit from the data and calculating the standard deviation gives the noise *B* and the SNR = *A*/*B*. The SNR decreases with shorter *t*_int_ [Figure 3(d)] to a value of 3.5 at *t*_int_ = 1 μs. We extrapolate the  trend for short *t*_int_ (dashed line) and estimate the minimum integration time *t*_min_ ≈ 1 × 10^–7^ s needed to achieve SNR = 1. This value compares well with state-of-the-art dispersive charge sensing of silicon QDs^5^ and is 4 orders of magnitude faster than previous dispersive charge sensing in bilayer graphene.^45^ Although the probe power used is close to optimal, more careful optimization could further enhance the readout performance.

Figure 3(e) shows the dependence of the SNR on resonator probe power (black dots). The signal strength grows with an increase in probe power up to a power of *P* = −115 dBm. Beyond this power level, the SNR remains constant. Additionally, when the power exceeds *P* = −95 dBm, thermal broadening becomes evident, marked by a significant increase in the peak width σ (orange squares).

Beyond charge sensing, we characterize the dipole coupling of the DQD to microwave photons in the resonator. The gate voltage *V*_L_ varies the energy detuning δ between charge states of an electron being in the left or in the right QD, forming a charge qubit with energy . At δ = 0, the states hybridize due to tunnel coupling *t*_c_ and are separated by the minimal charge qubit energy 2*t*_c_. Its interaction with the resonator depends on two properties of the charge qubit: The coupling strength is proportional to its electric dipole moment which is maximized when the electron wave function is completely delocalized between the QDs at δ = 0. Furthermore, the charge qubit needs to be resonant with the resonator (*E*_q_/*h* = *f*_r_) for the two systems to exchange energy, otherwise the interaction is dispersive.

Input-output theory^46^ describes photons in a resonator interacting with a two-level system. A comparison between resonator spectroscopy data and input-output theory characterizes the hybrid system and allows us to extract its coupling and decoherence properties (see also Supporting Information). We determine some system parameters from independent measurements, such as the frequency *f*_r_ and the line width κ of the unperturbed resonator. Parameters to be derived from input-output theory are the charge qubit decoherence rate γ and the DQD tunnel coupling *t*_c_. We also leave the coupling strength *g* as a fitting parameter that we can compare to the estimate . This value for *g* uses the lever arm difference β ≈ 0.037 ± 0.005 of the resonator gate *V*_R_ to the DQD that we estimate from finite bias measurements.

Figure 4(a) and (b) contrast Δϕ as a function of probe frequency *f*_p_ and detuning δ for two different interdot transitions that demonstrate qualitatively different behavior. The data displayed in Figure 4(a) is from the transition in Figure 3(a). Pink and blue marks indicate the line traces from Figure 3(b) taken at two different detunings. We interpret the observed resonator shift to lower frequencies around δ = 0 as the effect of the charge qubit with its minimal excitation frequency 2*t*_c_/*h* being larger than the resonator frequency *f*_r_. For 2*t*_c_/*h* ≫ *f*_r_, the interaction is dispersive and acts as a perturbation on the two subsystems. Dipole coupling to the charge qubit shifts the resonator frequency by . The black dashed line in Figure 4(a) indicates the cut for fixed probe frequency shown in panel (c). From fits of the transmission spectrum at each value of δ we extract the frequency shift Δ*f*_r_ as a function of detuning [panel (e)]. The largest shift is Δ*f*_r_ = −0.65 MHz at δ = 0. A least-squares fit of input-output theory to the full data gives best estimates of the free parameters (see Supporting Information for details). For the dispersive case with 2*t*_c_/*h* ≫ *f*_r_ the best fit is for *g*/2π = 49.7 MHz, γ/2π = 643 MHz and 2*t*_c_/*h* = 10.2 GHz. Solid lines in panels (c) and (e) overlay the data with Δϕ and Δ*f*_r_ calculated using this set of parameters, demonstrating reasonable agreement.

**Figure 4:**
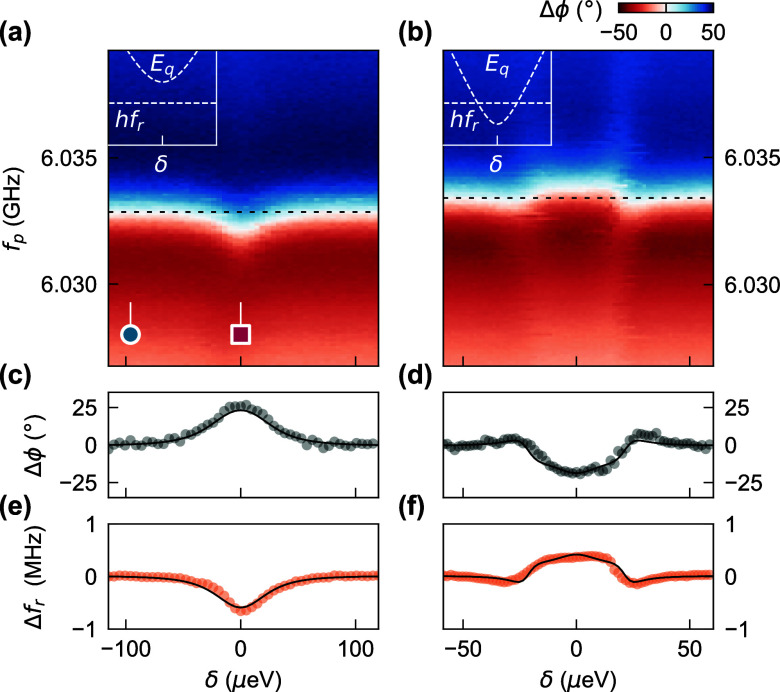
Comparison of charge–photon coupling for two different interdot transitions with 2*t*/*h* > *f*_r_ (left column) and 2*t*/*h* < *f*_r_ (right column). (a) Resonator phase signal as a function of DQD detuning δ and probe frequency *f*_p_. Dispersive interaction with the charge qubit shifts the resonator frequency around δ = 0. Pink and blue marks indicate the line traces shown in Figure 3 (b). (c) Line cut of Δϕ along detuning for fixed *f*_p_ indicated by the dashed black line in panel (a). (e) Extracted resonator frequency shift Δ*f*_r_ as a function of detuning. Solid black lines overlay input–output theory calculations for best fit parameters (*g*/2π, γ/2π, 2*t*/*h*) = (49.7 MHz, 643 MHz, 10.2 GHz). The spectroscopy data in (b) shows a different interdot transition with 2*t*/*h* < *f*_r_. A larger charge rearrangement was corrected in this data set. (d) Phase shift along the dashed black line in (b) and (f) resonator frequency shift extracted from (b). Calculations with best fit parameters (*g*/2π, γ/2π, 2*t*/*h*) = (37.5 MHz, 1.11 GHz, 2.99 GHz) are shown as solid black lines in (d) and (f).

The second data set [Figure 4(b)] was taken for a different interdot transition at different plunger gate voltages. The black dashed line indicates where the trace of Δϕ shown in panel (d) was taken, and we again extract the resonator shift Δ*f*_r_ as a function of detuning [panel (f)]. Both Δϕ and Δ*f*_r_ showcase the qualitative differences to the interdot transition in the left column of Figure 4. In contrast to the dispersive case, the charge qubit frequency crosses the resonator at δ = ± 25 μeV and reaches a minimal frequency 2*t*_c_/*h* < *f*_r_. Within this range of detuning, *E*_q_(δ)/*h* is below the resonator frequency *f*_r_ so that interactions with the charge qubit shift the resonator to higher frequencies by Δ*f*_r_ = 0.39 MHz. At the detuning energy where *E*_q_(δ)/*h* is close to *f*_r_, the interaction is resonant and the high decoherence rate γ of the charge qubit reduces the resonator quality factor. We determine the best fitting parameters for the 2*t*_c_/*h* < *f*_r_ case to be *g*/2π = 37.5 MHz, γ/2π = 1.11 GHz and 2*t*_c_/*h* = 2.99 GHz.

In both the dispersive and the resonant case we obtain a value for *g*/2π that is close to or even higher than our first estimate of 38 MHz. This confirms that there is no significant loss in coupling strength from impedance mismatches along the gate connected to the resonator. Differences to the estimated coupling strength are likely introduced through the high uncertainty in estimating the lever arm difference β from finite bias measurements. While the gate capacitances that determine the lever arms depend on the exact tuning of the DQD, they are mostly determined by the implemented gate layout. Generally, we also note that the charge qubit decoherence rate γ is large compared to the coupling strength *g* and resonator line width κ, preventing the system from reaching the strong coupling regime. We assume that the observed γ is large because the DQD is not sufficiently isolated from the leads.^6^ High tunnelling rates to the leads limit the average time charges spend in the DQD and act as a loss of charge qubit coherence. Effectively, the value for γ estimated from our measurements therefore sets an upper bound for the decoherence rate of a charge qubit in bilayer graphene. Overall, we observe a limited tuning range of tunnelling rates in this specific device due to accumulation of spurious dots under the barriers, although tunability and low tunnelling rates are generally achieved in BLG QD devices.^28,33,52^

We demonstrated electric dipole coupling of a bilayer graphene DQD to a high-impedance superconducting microwave resonator. We used the on-chip resonator for dispersive sensing and observed a signal-to-noise ratio well above unity even for integration times shorter than 1 μs. Furthermore, we investigated the charge-photon coupling in the dispersive and resonant cases. By comparing spectroscopy data to input-output theory, we estimated the relevant coupling parameters. Notably, the achieved coupling strengths are comparable to those in early stage silicon quantum dots,^13,53^ despite the small gate lever arms in our device. Our analysis shows that, with *g* ≫ κ already satisfied, only the charge qubit decoherence γ prevents the system from reaching the anticipated strong charge-photon coupling regime. Better tunability of tunnelling barriers and a reduction in charge decoherence should be achievable by an improved gate design.^4^

Our results demonstrate how hybrid cQED techniques can be used to probe bilayer graphene QDs and lead the way to coherent charge-photon coupling. To go further, hybridization of charge and spin degrees of freedom, achieved by similar means as in silicon,^12,54^ can enable strong spin-photon coupling also with bilayer graphene QDs. Looking ahead to spin and valley qubits, cQED applications such as fast state readout or long-range interactions may become essential for bilayer graphene as a material platform for qubits.

## Supporting Information Available

Additional details on device fabrication and device characteristics, input–output theory, and fitting procedure (PDF)
