# Supplementary material for: Electric Dipole Coupling of a Bilayer Graphene Quantum Dot to a High-Impedance Microwave Resonator
Source: Nano Lett. 2024 Jun 4;24(24):7508–14. doi: 10.1021/acs.nanolett.4c01791 (PMC11194813; doi:10.1021/acs.nanolett.4c01791)
Supplement: Supplementary file 1 — nl4c01791_si_001.pdf [file nl4c01791_si_001.pdf]

Supporting Information: Electric dipole coupling of a bilayer graphene quantum dot to a high-impedance microwave resonator

Max J. Ruckriegel,[*,†] Lisa M. Gächter,[†] David Kealhofer,[†] Mohsen Bahrami Panah,[†,‡] Chuyao Tong,[†] Christoph Adam,[†] Michele Masseroni,[†] Hadrien Duprez,[†] Rebekka Garreis,[†] Kenji Watanabe,[¶] Takashi Taniguchi,[§] Andreas Wallraff,[†,‡] Thomas Ihn,[†,‡] Klaus Ensslin,[†,‡] and Wei Wister Huang[†]

†*Laboratory for Solid State Physics, ETH Zürich, CH-8093 Zürich, Switzerland*

‡*Quantum Center, ETH Zürich, CH-8093 Zürich, Switzerland*

¶*Research Center for Electronic and Optical Materials, National Institute for Materials Science, 1-1 Namiki, Tsukuba 305-0044, Japan*

§*Research Center for Materials Nanoarchitectonics, National Institute for Materials Science, 1-1 Namiki, Tsukuba 305-0044, Japan*

E-mail: maxr@phys.ethz.ch

Device Fabrication

The sputtering process was carried out in an Orion 8 magnetron sputtering system (AJA International, Inc.) in reactive dc mode from a NbTi (Nb/Ti 70/30 wt%) target (ACI Alloys, Inc.) with 99.99% purity at 1.5×10^{-8} mTorr base pressure. We initiate the process with a target cleaning step (5 min exposure to argon (Ar) plasma at 50 sccm flow rate and 100 W) and a conditioning phase (4 sccm nitrogen (N_2) for 1 min with shutter closed) . The subsequent deposition stage consists of Ar/N_2 flows at 50 sccm and 4 sccm, respectively, maintaining a power of 100 W, a pressure of 3.5 mTorr, and a working distance of 10 cm. The parameters were chosen to optimize the growth conditions for NbTiN films, film quality, thickness uniformity, and structural properties.

We sputter a nominally 15 nm thick film of NbTiN on a two-inch wafer of intrinsic silicon ($\rho > 10\,\mathrm{k\Omega\,cm}$) with 100 nm of thermally grown SiO_2 (Alineason Materials Technology GmbH). The microwave circuit is patterned using a direct-write photo-lithography system (Heidelberg Instruments DWL66+) and reactive ion etching (RIE) with SF_6/Ar. Afterwards, we deposit gold markers and bondpads in a lift-off process and dice the wafer into 5×8 mm chips. The inductance per unit length L_l of the coplanar waveguide resonator is dominated by the large contribution of the sheet kinetic inductance of the NbTiN film. From the resonator dimensions (width 1.6 µm, gap to ground 8.6 µm and length 900 µm) and its center frequency $f_\mathrm{r} = 6.033\,\mathrm{GHz}$ we estimate the film's sheet kinetic inductance to be $L_{\square,\mathrm{kin}} \approx 150\,\mathrm{pH}/\square$. The on-chip low-pass filters on all gate lines are formed by a shunt capacitance to ground $C_\mathrm{f} \approx 0.2\,\mathrm{fF}$ in series with the inductance $L_\mathrm{f} \approx 210\,\mathrm{nH}$ of a 2 µm wide NbTiN wire. This results in a filter cut-off frequency of $f_\mathrm{c} = (2\pi\sqrt{L_\mathrm{f}C_\mathrm{f}})^{-1} \approx 0.78\,\mathrm{GHz}$.

The vdW material stack is fabricated on the pre-patterned chips using standard mechanical exfoliation from bulk crystals and polymer-based dry transfer techniques. We first deposit the bottom hBN ($\sim$ 25 nm thick) and graphite back-gate onto the chip and clean it from polymer residues. The top hBN ($\sim$ 35 nm thick) and bilayer graphene are placed onto the pre-deposited bottom half in a separate deposition. Metal gates are fabricated in

a lift-off process by electron beam lithography (EBL) and metal evaporation. For ohmic contacts to the graphene, we etch hBN before metal deposition using RIE with CHF_3. The split-gates are made from 3/20 nm Cr/Au with a 100 nm wide channel. Plunger and barrier gates are also made from 3/20 nm Cr/Au with 25 nm in width and a gate pitch of 70 nm. They are separated from the first gate layer by 20 nm of Al_2O_3 grown by atomic layer deposition (ALD) at 150 °C. The Al_2O_3 layer is removed by wet etching to establish contact to the resonator below the dielectric.

Input-Output Theory

The complex transmission S_{21} through the microwave feedline with a resonator coupled in a notch-type configuration can be derived from input-output theory[1] to be

$$S_{21}(f_\mathrm{p}, \delta) = 1 + \frac{i\kappa_\mathrm{ext}}{2\pi(f_\mathrm{p} - f_\mathrm{r}) - i\kappa + g^* \chi_\mathrm{e}}.$$

The resonator frequency f_r and total linewidth $\kappa = \kappa_\mathrm{ext} + \kappa_\mathrm{int}$ are determined from a fit to the spectrum of the unperturbed resonator.[2] For this fit, the external coupling κ_ext is taken as a complex-valued parameter to account for an asymmetric Fano lineshape of the resonance arising from non-idealities in the resonator-feedline coupling.

The effect of the charge qubit is described by its electric susceptibility

$$\chi_\mathrm{e} = \frac{g^*}{i\gamma + 2\pi(f_\mathrm{p} - E_\mathrm{q}(\delta)/h)},$$

that considers the charge qubit decoherence rate γ and probe detuning. The charge qubit energy $E_\mathrm{q}(\delta) = \sqrt{4t_\mathrm{c}^2 + \delta^2}$ depends on the interdot tunnel coupling t_c and the DQD energy detuning δ. We calculate δ from the lever arms $\alpha^\mathrm{L}_\mathrm{dL(R)}$ of the left plunger gate to the left (right) dot as $\delta = (\alpha^\mathrm{L}_\mathrm{dL} - \alpha^\mathrm{L}_\mathrm{dR})\mathrm{e}V_\mathrm{L} = 0.024 \times \mathrm{e}V_\mathrm{L}$ with the lever arms determined by finite bias measurements of the DQD. The effective charge qubit coupling strength is $g^* = g\frac{2t_\mathrm{c}}{E_\mathrm{q}(\delta)}$

using the bare resonator coupling strength

$$g/2\pi = \frac{\sqrt{\pi}}{2}\beta f_{\mathrm{r}}\sqrt{\frac{Z_{\mathrm{r}}}{h/e^2}}.$$

It is proportional to the lever arm difference of the coupling gate $\beta = \alpha^{\mathrm{R}}_{\mathrm{dR}} - \alpha^{\mathrm{R}}_{\mathrm{dL}}$, the resonator frequency f_{r}, and the square root of the resonator impedance Z_{r}.

Fitting procedure

The parameters g, γ and $2t_{\mathrm{c}}$ are determined by a least-squares fitting routine. We calculate the complex transmission $S_{21}(f_{\mathrm{p}}, \delta)$ from input-output theory for a given set of fit parameters $\theta = [g, \gamma, 2t_{\mathrm{c}}]$ and compute the mean squared error $Q(\theta)$ between calculation and measurement data. The parameters given in the main text for each data set are the parameters $\hat{\theta}$ that minimize the error, found by sampling all three parameters over a wide range of values. We numerically calculate the hessian matrix $H_{i,j} = \frac{N}{Q(\hat{\theta})}\frac{\partial^2 Q(\theta)}{\partial\theta_i\partial\theta_j}\Big|_{\hat{\theta}}$ around $\hat{\theta}$ with N the size of S_{21}. From H we calculate the variance-covariance matrix $K = H^{-1}$ which contains information about uncertainties and correlations of our parameter estimates. For the dispersive case with $2t_c/h \gg f_r$ the parameters that minimize the mean squared error are $g/2\pi = 49.72 \pm 0.27\,\mathrm{MHz}$, $\gamma/2\pi = 643 \pm 24\,\mathrm{MHz}$ and $2t_{\mathrm{c}}/h = 10.193 \pm 0.046\,\mathrm{GHz}$. The strongest correlation is observed between g and $2t_{\mathrm{c}}$ as expected from the definition of the effective coupling strength g^* above, while the estimate of γ is mostly uncorrelated to the other two parameters. The relative uncertainty in our estimate of γ is significantly larger compared to the uncertainty in the other two parameters. This is because the resonator-qubit detuning is large over the whole range of δ. The incoherent dispersive interaction dominates the resonator response and does not depend strongly on the decoherence rate γ, rendering the mean squared error insensitive to the estimate of the decoherence rate.

For the resonant case with $2t_c/h < f_r$ the parameters that minimize the mean squared error are $g/2\pi = 37.49 \pm 0.19\,\mathrm{MHz}$, $\gamma/2\pi = 1.112 \pm 0.008\,\mathrm{GHz}$ and $2t_{\mathrm{c}}/h = 2.986 \pm 0.015\,\mathrm{GHz}$.

We observe stronger correlations between each pair of parameters compared to the dispersive case. Furthermore, the uncertainty in γ is smaller, because the resonant interaction is limited by charge qubit decoherence. Therefore, the mean squared error is more sensitive to the estimate of the decoherence rate.

References

(1) Burkard, G.; Petta, J. R. Dispersive readout of valley splittings in cavity-coupled silicon quantum dots. *Phys. Rev. B* **2016**, *94*, 195305.

(2) Probst, S.; Song, F. B.; Bushev, P. A.; Ustinov, A. V.; Weides, M. Efficient and robust analysis of complex scattering data under noise in microwave resonators. *Review of Scientific Instruments* **2015**, *86*, 024706.
